# Psychophysiological Markers of Vulnerability to Psychopathology in Men with an Extra X Chromosome (XXY)

**DOI:** 10.1371/journal.pone.0020292

**Published:** 2011-05-31

**Authors:** Sophie van Rijn, Hanna Swaab, Maurice Magnée, Herman van Engeland, Chantal Kemner

**Affiliations:** 1 Clinical Child and Adolescent Studies, Leiden University, Leiden, The Netherlands; 2 Leiden Institute for Brain and Cognition, University Medical Center Leiden, Leiden, The Netherlands; 3 Rudolf Magnus Institute of Neuroscience, Department of Psychiatry, University Medical Center Utrecht, Utrecht, The Netherlands; The University of Hong Kong, Hong Kong

## Abstract

Studying genetically defined syndromes associated with increased risk for psychopathology may help in understanding neurodevelopmental mechanisms related to risk for psychopathology. Klinefelter syndrome (47,XXY) is one of the most common sex chromosomal aneuploidies (1 in 650 male births) and associated with increased vulnerability for psychopathology, including psychotic symptoms. Yet, it remains unknown whether this increased risk is associated with underlying psychophysiological mechanisms that are typically deficient in individuals with psychotic disorders. The present study assessed three “classic” psychophysiological markers of psychosis in Klinefelter syndrome (KS): smooth pursuit eye movements (SPEM), prepulse inhibition (PPI) and P50 suppression. Fourteen adults with KS and 15 non-clinical adults participated in the study. Data on SPEM (reflecting visuo-motor control) as well as PPI and P50 suppression (reflecting sensory gating) were collected. Dysfunctions in SPEM were observed in individuals with KS, with less smooth pursuit as expressed in lower position gain. Also, reduced sensory gating in individuals with KS was suggested by significantly reduced prepulse inhibition of the startle response (PPI) (effect size 1.6). No abnormalities were found in suppression of the P50 (effect size 0.6). We speculate that impairments in these psychophysiological mechanisms may reflect core brain dysfunctions that may also mediate the described increased vulnerability for psychotic symptoms in KS. Although speculative, such *deficit* specific, rather than *disorder* specific, psychophysiological dysfunctions in KS might convey vulnerability to other types of psychopathology as well. As KS already can be diagnosed prenatally, the predictive value of childhood impairments in prepulse inhibition and smooth pursuit for development of psychopathology later in life could be assessed. In sum, studying individuals with KS may prove to be an avenue of research leading to new hypotheses and insights into “at risk” pathways to psychopathology.

## Introduction

Studying genetically defined syndromes associated with increased risk for psychopathology may help in understanding developmental pathways to psychopathology. The strength of such studies lies in the opportunity to study the bottom-up effects of a developmental condition of prenatal origin, thereby complementing top-down studies where study populations are defined based on behavioural outcome. In this regard, a relevant genetic condition is Klinefelter syndrome, characterized by the presence of an additional X chromosome in boys and men. Among the neurodevelopmental risks, increased risk for psychotic symptoms has been documented.

Studies into vulnerability for psychotic disorders have been driven by the notion that the X chromosome is enriched with genes involved in neural development and related cognitive and mental functioning. These studies were also driven by reports of an increased prevalence of the XXY pattern among patients with schizophrenia as compared to the general population [1]. Similarly, among children diagnosed with childhood onset schizophrenia (COS), 2 out of 66 were found to have the XXY pattern [2], which is higher than expected based on the prevalence of XXY in the general population (1 in 650 liveborn boys). There are several studies on risk for psychotic symptoms and psychotic disorders in Klinefelter syndrome. A screening of hospital discharges revealed an increased relative risk (4.7 times more often) for being hospitalized with a psychotic disorder for individuals with XXY (n = 832) as compared to individuals with the typical XY pattern (n = 4033) [3]. In line with these data, a study on schizotypal traits and clinical schizophrenia symptoms (including psychotic symptoms) in XXY adults, symptom scores were significantly increased across all domains of the schizophrenia spectrum, with effect sizes (cohen's d) ranging from 1.4 to 1.8 [4]. A psychiatric screening of 31 of these adults with XXY revealed diagnoses of schizophrenia (n = 1), bipolar disorder (n = 2) and delusional disorder (n = 1) [5], with the prevalence of psychotic disorders higher as compared to men in the general population. Not only in adults, but also in children with XXY an increased risk for psychotic symptoms has been observed. Bruining et al. [6] have reported on a psychiatric screening of a childhood XXY sample (n = 51), with two subgroups: one subgroup was recruited through active follow-up of prenatally diagnosed children whereas the other subgroup was diagnosed postnatally and recruited with help of pediatricians, endocrinologists and support groups. Their data showed that 8% met criteria for a psychotic disorder and 45% had isolated psychotic symptoms. There were no significant differences between the two subgroups with regard to risk for psychopathology.

These findings of increased risk for psychotic disorders calls for research into the biological and psychological mechanisms driving this increased risk, in order to gain insight into ‘at risk’ pathways to psychosis. As there is variance in the clinical phenotype in KS and not all individuals develop psychotic symptoms, KS may be considered a ‘high risk’ population which can be studied to identify risk parameters. One hypothesis is that individuals with Klinefelter syndrome and individuals with psychotic disorders might share some of the underlying brain dysfunctions that play a role in the development of psychotic or schizotypal symptoms. Such mediating mechanisms at the level of brain morphology and brain function are in between the genotype en clinical phenotype, and referred to as endophenotypes. Endophenotypes of psychosis are present in both patients with psychotic disorders and (healthy) individuals at high genetic risk for the disease [7].

Among the most consistently reported and most often replicated endophenotypes in psychosis are deficits in eye tracking abnormalities and sensory gating [7], [8]. Eye tracking dysfunction, i.e. smooth pursuit eye movements (SPEM), is a well known endophenotype associated with psychotic disorders [9]. Abnormalities in smooth pursuit of moving visual input, as indicated by lower position gain or more saccades, point to basic visuomotor dysfunction in the brain. Sensory gating refers to a basic inhibitory process in the brain regulating the input of sensory information. This filtering mechanism gates out irrelevant or repetitive information in such a way that brain systems are not flooded with information. There are two classic paradigms for measuring sensory gating, prepulse inhibition (PPI) and P50 suppression, although these may tap into different aspects of gating as indicated by differences in the neural systems involved [10] and lack of strong correlations between PPI and P50 [11]. In the prepulse inhibition (PPI) paradigm, the magnitude of the eyeblink response is suppressed when the acoustic startle stimulus is preceded by a weaker, prepulse, stimulus. In the P50 paradigm, the degree of sensory gating is reflected in event related potentials (ERP's) evoked by two identical clicks in close succession. The degree of suppression of the ERP response to the second click, more specifically the positive peak after 50 milliseconds (P50), indicates the degree of sensory gating. Both SPEM dysfunctions and sensory gating deficits are consistently found in individuals with psychotic disorders [12]–[14] and appear to be genetically determined rather than due to the clinical presence of the disease or due to medication ([15]–[20], but see [21]).

The present study is the first to investigate smooth pursuit eyemovements (SPEM) and sensory gating, as measured in PPI and P50 suppression, in individuals with Klinefelter syndrome. Our hypothesis was that smooth pursuit and sensory gating may be atypical in this ‘high risk’ population. If so, this knowledge might contribute to our understanding of common gene-brain-behavior pathways to psychotic symptoms in a genetic disorder, Klinefelter syndrome, and behaviorally defined disorders such as psychotic disorders. Hence, this knowledge may provide insight in gene-brain behavior mechanisms that are *deficit* specific rather than *disorder* specific.

## Methods

### Ethics statement

The study was approved by the local ethical board of the University Medical Center Utrecht (Utrecht, The Netherlands) and after complete description of the study to the subjects, written informed consent was obtained according to the declaration of Helsinki.

### Subjects

14 XXY men and 15 non-clinical control men participated in the study. XXY men were recruited with help from the Dutch Klinefelter Association, and were not selected for psychological, behavioral or cognitive abnormalities. Diagnosis of Klinefelter syndrome was confirmed by genetic analysis (i.e. karyotyping) using standard procedures. All, except one, of the XXY men were treated with testosterone supplements. Controls were recruited using advertisements in local newspapers or were drawn from a database in our department. Intellectual functioning, measured with the WAIS-IV, was assessed in both groups. For group characteristics, see table 1.

**Table 1:** Age and intellectual functioning (mean, SD) in the Klinefelter group as compared to the non-clinical control group.

	Control (n = 15)	Klinefelter (n = 14)	Statistics
**Age (mean, SD)**	23.9 (5.7)	30.0 (8.4)	F(1,27) = 5.9, p = 0.02
**FSIQ (mean, SD)**	108.8 (15.1)	89.6 (13.3)	F(1,27) = 13.5, p<0.01
**VIQ (mean, SD)**	109.4 (15.9)	93.6 (12.6)	F(1,27) = 8.7, p<0.01
**PIQ (mean, SD)**	105.4 (17.4)	90.7 (12.5)	F(1,27) = 6.6, p = 0.01

FSIQ: full scale IQ, VIQ: verbal IQ, PIQ: performance IQ.

### Recordings

Recordings were obtained from 32 AgAgCl electrodes using a BioSemi Active Two EEG system (Biosemi, Amsterdam). For the P50 measurement, EEG was sampled at 2048 Hz, referenced to an additional active electrode (Common Mode Sense) during recording, and stored as a continuous signal. An electrode placed on the left mastoid was used as off-line reference for EEG measurement. For PPI, electromyographic (EMG) activity of the right orbicularis oculi muscle was recorded from bipolar electrodes. One was placed over the medial aspect of the muscle and one displaced 0.5 cm laterally. Horizontal and vertical eye movements were recorded using electro-oculography (EOG) to obtain SPEM information. All data were analysed using the software package Brain Vision Analyzer (Brain Products, München). All signals were digitized online at a rate of 500 Hz and stored as a continuous signal.

### Smooth pursuit paradigm

The subject sat upright in a dentist-chair and the head was held steady using a vacuum-cushion, which reduced head movements. Stimuli were displayed on a 21-inch computer screen (42 by 32 cm), which was positioned 1 meter in front of the subject's eyes. The display size was 800 by 600 pixels. Eye movements were recorded using electro-oculography (EOG) by means of Psylab hardware and software provided by Contact Precision Instruments (London, UK), filtered online (fixed built-in bandpass filter of 0.01–700 Hz), and sampled at 500 Hz. The tin electrodes were placed supraorbitally and infraorbitally over the left eye and at the outer canthi of both eyes. The ground electrode was placed on the forehead.

For the smooth pursuit task, the target was a small, but clearly visible, white moving dot (2 by 2 pixels) on a uniform dark-gray background. There were seven trials, each consisting of 5 movements of the dot from left to right and back again with amplitude from left to right of 20 degrees of visual angle. In each trial the dot moved at a constant velocity (sinusoidal motion). Stimulus velocities of 8, 13, 16, 20, 24, 29, and 35 degrees per second (deg/s) were used, and in this order. For training purposes, 2 practice trials were included. After filtering the horizontal electro-oculography (HEOG) signal (low pass filter at 15 Hz), a calibration factor was obtained from the trial with lowest target frequency. The velocity was determined from the calibrated signal following a previously validated method [22]. For each sample point the velocity of the tracking was calculated by subtracting the position value at 10 msec before the given point from the position value at 10 msec after the given point and dividing the result by 20 msec. Saccadic onsets and offsets were then determined by a computer program, and only if the calibrated signal between onset and offset differed more than 0.5 deg, they were taken to mark a saccade. Saccades are defined as a period of absolute velocity above 35 deg/sec between two successive acceleration peaks of opposite sign. To find the onset and offset points of a saccade, first peaks of acceleration in the velocity pattern were determined with an absolute value over 200 deg/sec. Velocity gain is defined as mean eye velocity divided by target velocity. Velocity gain was determined from points that were not marked as saccades and for which the target was at least 5 degrees away from the extremities where it changed its direction. The relevant variables in this paradigm are the number of saccades per second and position gain at seven levels of velocity. Since values for the position gain and the saccade parameters were determined from a HEOG signal, no absolute position of gaze information was available, so it was not possible to determine saccadic type (anticipatory, leading, catch-up, etc).

### Prepulse inhibition paradigm

The startle test consisted of a block of 24 randomized trials: 12 trials consisted of a startle stimulus preceded by a prepulse stimulus (prepulse trials) and 12 trials consisted of a startle stimulus alone (pulse alone trials). The intertrial intervals were randomised between 12 and 23 s. The prepulse and startle stimuli were bursts of white noise (duration 25 and 30 ms, intensity 87 dB and 107 dB, respectively) over a 30 dB background noise, with a fixed interstimulus interval of 130 ms (prepulse onset to pulse onset). The stimuli were presented binaurally through stereo insert earphones (Eartone ABR).

EMG data were filtered offline with a high-pass filter of 30 Hz and a low-pass-filter of 200 Hz. Epochs from −50 ms pre-stimulus until 200 ms post-stimulus were extracted from the continuous data, and the baseline was corrected using the data for 50 ms prior to stimulus-onset. Thereafter, the data were rectified. Last, assessment of the maximal peak amplitude and PPI quantification took place within a window of 20–100 ms after stimulus onset. PPI was defined as the percentage reduction in startle magnitude of prepulse-pulse trials compared to the pulse alone trials (PPI = 100(1-pp/p), where pp indicates amplitude over prepulse-pulse trials and p indicates amplitude over pulse alone trials.

### P50 gating paradigm

The auditory task two auditory stimuli were presented with an interstimulus interval of 500 ms. Stimuli were 36 click pairs, consisting of white noise bursts of 1.5 ms, with an intensity of 86 dBa. The intertrial interval was 10 s. The auditory stimuli were presented binaurally through stereo insert earphones (Eartone ABR). Individuals were instructed to count the stimulus pairs, and reported this afterwards.

After recording, the EEG and EOG signals were offline filtered between 1 Hz and 50 Hz (slope 24 dB/oct) and segmented into epochs at an interval between 100 ms prestimulus and 400 ms poststimulus. After EOG correction [23], all epochs were corrected for artifacts and baseline. ERPs were averaged separately for conditioning and testing stimuli.

P50 were taken from the Cz electrode and scored according to Nagamoto et al. [24]. P50 suppression was defined as the ratio *T*/*C*, where *T* represents the mean P50 amplitude to the testing stimulus (preceded by either auditory or visual conditioning stimuli), and *C* represents the P50 amplitude to the auditory conditioning stimulus only. N1 peaks were measured as the greatest negativity in a window 90–160 ms poststimulus.

### Statistical analyses

Group differences in T/C ratios obtained in the P50 gating paradigm were tested using multivariate analyses of variance. The percentages prepulse inhibition in the Klinefelter group were compared to those in the control group using multivariate analyses of variance. For the smooth pursuit paradigm, two analyses of variance (repeated measures) were carried out; one for the position gain and one for the number of saccades, with one between-factor ‘Group’ with two levels (XXY versus control) and one within-factor ‘stimulus velocity’ with seven levels (8 to 35 deg/s). For all analyses, age and IQ were entered as covariates in multivariate analysis of variance used for testing group effects. Effect sizes were calculated as cohen's d (absolute difference between the groups divided by the mean standard deviation of the control group).

## Results

### Smooth pursuit

**Figure 1:**
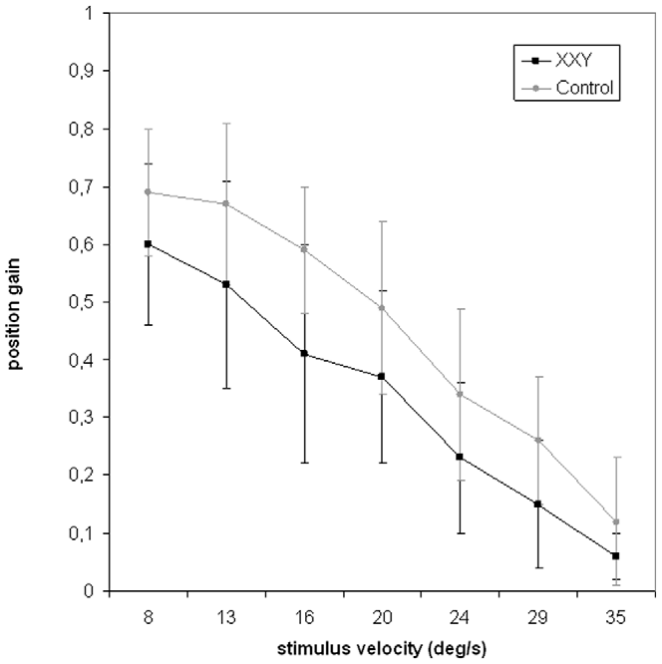
Smooth pursuit position gain as a function of stimulus velocity. A significantly lower position gain was found in the XXY group as compared to controls.

Two men with XXY did not fully complete this test and their data were not included in the analyses. There was a significant main effect of group on position gain (F(1,25) = 4.9, p = 0.03), with overall lower position gain in the XXY group (see figure 1), indicating less smooth pursuit in the XXY group. There was no significant group by stimulus velocity interaction, indicating that the reduction in smooth pursuit did not deteriorate more with increasing speed of target in the XXY group. Also, there was no significant main effect of age or IQ.

**Figure 2:**
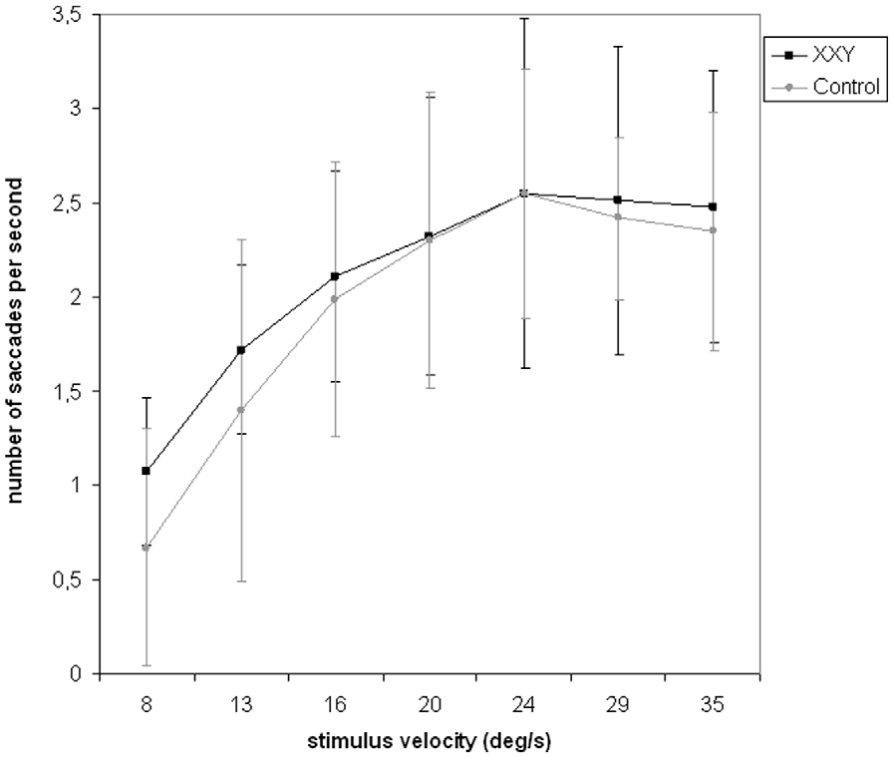
Number of saccades as a function of stimulus velocity. No significant group effects or interactions were observed.

Besides position gain, we also analyzed number of saccades as a function on increasing stimulus velocity. Repeated measures analysis did not show a significant main effect of group, age or IQ. Also, there was no significant group by stimulus velocity interaction. See figure 2.

### Prepulse inhibition

Due to incomplete data and technical errors, data from 3 control subjects and 1 XXY subject could not be included in the analyses.

**Figure 3:**
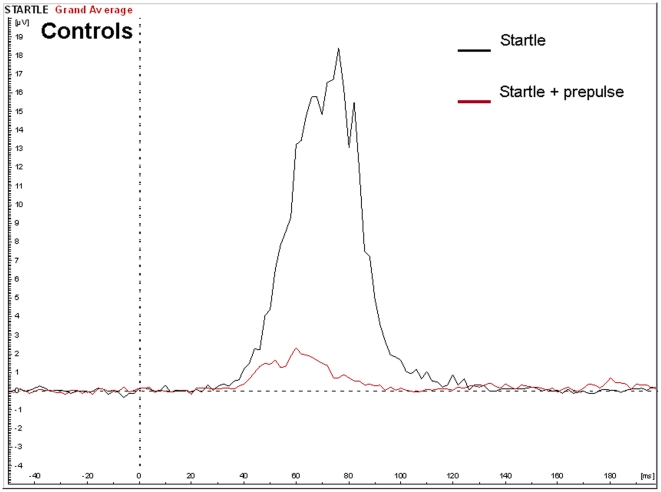
EMG potentials in the control group in response to the startle with and without a prepulse in the PPI paradigm.

**Figure 4:**
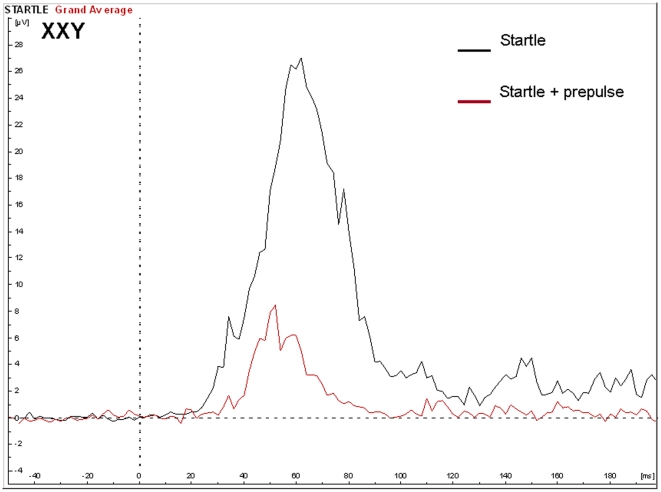
EMG potentials in the XXY group in response to the startle with and without a prepulse in the PPI paradigm.

There was a main effect of group, with the percentage prepulse inhibition (PPI) significantly lower in the Klinefelter group (71%, SD 19.9%) as compared to the control group (86%, SD 9.1%), F(1,23) = 6.5, p = 0.016. The effects size was 1.6. There was no significant main effect of age or IQ. See figures 3 and 4 for averaged signals.

### P50 gating

**Figure 5:**
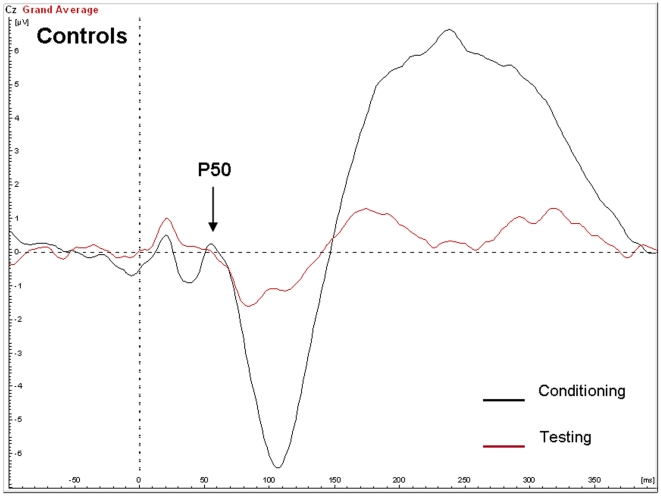
Auditory evoked potentials in the control group in response to the conditioning and testing stimulus in the P50 gating paradigm.

**Figure 6:**
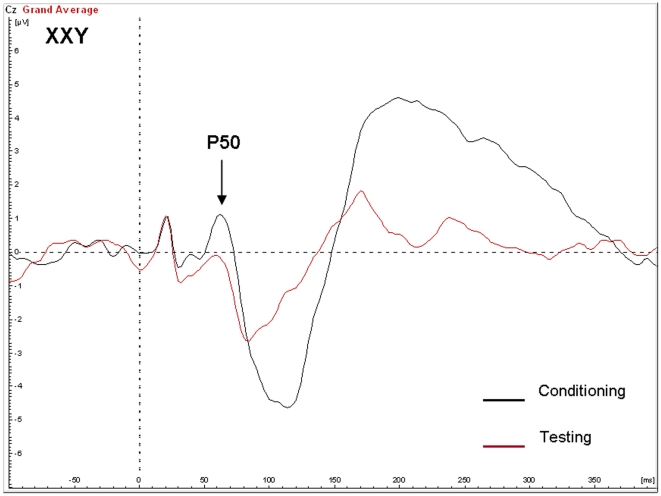
Auditory evoked potentials in the XXY group in response to the conditioning and testing stimulus in the P50 gating paradigm.

One subject in the Klinefelter group showed a T/C ratio at P50 of 3.5 standard deviations above group mean and was therefore excluded from analysis of P50 peaks. There was no significant main effect of group on suppression of the P50 or N100 (see figures 5 and 6 for averaged signals). In other words, there was no difference between the mean T/C ratio at P50 in the Klinefelter group (0.40, SD 0.35) as compared to the control group (0.62, SD 0.99), F(1,26) = 0.56, p = 0.46. Although not significant, the effect size (cohen's d) was 0.63, i.e. the distance between the group means was 0.63 standard deviations. Also, there was no significant main effect of age or IQ.

## Discussion

This is the first study to investigate whether Klinefelter syndrome (KS) is associated with impairments in smooth pursuit eye movements (SPEM), prepulse inhibition (PPI) or P50 suppression, psychophysiological dysfunctions that are considered reliable markers of liability to psychotic disorders. Indeed, dysfunctions in smooth pursuit eye movements were observed in individuals with KS, with less smooth pursuit as compared to controls. Also, reduced prepulse inhibition of the startle response (PPI) was found in individuals with KS, suggesting reduced sensory gating. No abnormalities were found in suppression of the P50.

Eyetracking dysfunction in individuals with KS involved less smooth pursuit of a visually moving target (expressed in smaller position gain), independent of stimulus velocity. This is likely to result in gaze progressively lagging behind the moving target. Smooth pursuit is one of two ways by which voluntarily shift in gaze is obtained, the other being saccadic eye movements. Saccades are rapid eye movements that serve to direct gaze rapidly from one point to another. A lower position gain may be accompanied by an increase in saccadic frequency to close the gap with a moving target [25]. Although the XXY group showed significantly lower position gain, a significantly higher number of saccades was not found. Although speculative, the absence of an increase in saccadic frequency as compared to controls, suggests that the XXY group may not have been able to compensate for the increasing gap with the moving target.

Reduced prepulse inhibition (PPI) of the startle response was also found in individuals with XXY. The degree of inhibition was 1.6 standard deviations below that of controls, revealing a large effect size on this measure. The reduction of the amplitude of startle reflects the ability of the nervous system to temporarily adapt to a strong sensory stimulus when a preceding weaker signal is given to warn. It has an adaptive function by filtering, or gating out, excess or trivial information. It has been postulated that such sensory gating helps to “regulate environmental inputs, to navigate successfully in a stimulus-laden world, and to selectively allocate attentional resources to salient stimuli” [26]. Hence, individuals with PPI deficits are prone to be flooded with information, resulting in sensory overload and cognitive fragmentation [27]. It will be interesting to examine in future and much larger studies, which aspects of the cognitive and behavioral phenotype of Klinefelter syndrome are related to PPI deficits. Although a deficiency in sensory gating was clearly indicated by the PPI results, we did not find significant abnormalities in P50 suppression. Although speculative, there may be several alternative interpretations of this finding: A) the P50 paradigm and the PPI paradigm may measure different aspects of sensory gating as has been suggested by others [10], [11] B) PPI is a more sensitive measure of sensory gating as compared to P50 suppression and C) the statistical power for finding group effects on P50 was too low in this study as indicated by a nonsignificant effect size (cohen's d) of 0.63.

Less smooth pursuit and prepulse inhibition deficits is also characteristic for patients with psychotic disorders [7]–[9], [12], [25], [28]–[31]. In line with findings in the XXY group, other populations with increased vulnerability for schizotypal or psychotic symptoms also display smooth pursuit eyetracking dysfunctions, ranging from individuals with high levels of schizotypal traits [32]–[36], relatives of patients with a psychotic disorder (for a review and meta analysis see [37]) and other genetic syndromes associated with increased risk for psychosis such as 22q11 (Velocardiofacial syndrome) [38]. Similarly, reduced PPI is also seen in these high risk populations, such as individuals with Schizotypal Personality Disorder [39], [40], relatives of patients with schizophrenia [40], [41] and, although to a lesser degree, in individuals from the general population with high levels of schizotypal traits (for a review, see [42]).

Based on these studies and others, it is thought that eye tracking deficits and sensory gating deficits are trait markers in psychotic disorders and can not be attributed to result from antipsychotic medication, mental state or other potential confounders. Our findings suggest that these traits markers are also present in individuals with Klinefelter syndrome. As sensory gating deficits are also found in other conditions, such as bipolar disorder [43], [44], obsessive compulsive disorder ([45], but see [46]), Huntington's disease [47], Tourette's syndrome [48], autism ([49], but see [50]) and Asperger syndrome [51], it would be interesting to assess whether prepulse inhibition (PPI) deficits in Klinefelter syndrome are also associated with increased risk for other types of psychopathology besides psychotic traits. In this respect, vulnerability for autism spectrum traits is of particular interest. In children and adults with Klinefelter syndrome, increased levels of autism traits have been reported [52], [53]. Also, increased risk for autism spectrum disorders (ASD) in children with Klinefelter syndrome has been observed, with 11% meeting criteria for ASD in a prenatal follow-up sample [54] and 27% meeting criteria for ASD in a sample that was a combination of referred cases and prenatal follow-up ([6], for case reports see [55]). Although speculative, deficient PPI might be a common mechanism underlying both autistic and psychotic traits in individuals with Klinefelter syndrome. Although this hypothesis is yet to be tested, common behavioral (for example [56]) and neurocognitive abnormalities (for example [57]) in both conditions support this idea. In Magnetic Resonance Imaging (MRI) studies comparing individuals with ASD and individuals with psychotic disorders, lower grey matter volumes within limbic-striato-thalamic circuitry [58] and abnormalities in the temporal lobe, cerebellum and striatum [59] were common to ASD and psychotic disorders. Interestingly, these common brain abnormalities at least partially overlap with the neural circuitry involved in PPI, which involves cortico-striatial-pallido-thalamic pathways based on animal studies [60] and regions such as the striatum, hippocampus, thalamus, and frontal and parietal cortical regions based on human studies [61]. All in all, it would be interesting to assess in future studies whether deficient PPI is a common mechanism underlying both autistic and psychotic traits in Klinefelter syndrome.

Among the limitations of this study are the relatively small sample sizes, which may have resulted in less statistical power. Also, groups were not optimally matched for intelligence and age, although this was corrected for in all statistical analyses. We included IQ differences in our analyses and did not find a main effect IQ on psychophysiological parameters. As IQ was a covariate, significant group effects were found after controlling for differences in IQ. Another limitation is that it was not possible to determine saccadic type (anticipatory, leading, catch-up, etc), since values for the position gain and the saccade parameters were determined from a HEOG signal.

In conclusion, we speculate that impairments in basic psychophysiological mechanisms in the area of visuo-motor control (SPEM) and sensory gating (PPI), reflect core brain dysfunctions that may also mediate the described increased vulnerability for psychotic symptoms in Klinefelter syndrome. These psychophysiological traits may be shared by Klinefelter syndrome and psychotic disorders, and may point to a final common pathway to liability for psychotic symptoms. Although speculative, such ‘deficit’ specific, rather than ‘disorder’ specific, psychophysiological dysfunctions may convey vulnerability to autistic symptoms as well. Our preliminary data call for substantially larger studies that allow for replication as well as correlational analysis between psychophysiological deficits and various clinical symptoms, not only psychotic symptoms but also autistic symptoms, as well as cognitive dysfunctions. Sensory gating involves (preattentive or controlled) attentional processes, which are found to be affected in Klinefelter syndrome among other executive functions [62]–[64]. It would therefore be interesting to investigate the relationship between sensory gating and attention regulation problems in Klinefelter syndrome. This would require a larger sample and manipulation of the PPI paradigm to include short versus long lead intervals as well as to-be-attended and to-be-ignored lead stimuli. In addition, a range of cognitive attention regulation tasks could help in relating attention problems to degree of sensory gating.

Future studies on Klinefelter syndrome may also take a developmental approach by studying if there is a predictive value of early eyetracking dysfunctions and sensory gating deficits for developing psychopathology later in development. As Klinefelter syndrome is a genetic disorder that can be identified as early as prenatally, there are opportunities to study these traits already very early in development. Risk for the development of psychopathology in Klinefelter syndrome during the course of development is probably mediated by a combination of genetic factors in interaction with non-genetic factors (hormones, parenting, stress, etc), which may also be addressed in future studies. Studying individuals with an extra X chromosome may prove to be an avenue of research leading to new hypotheses and insights into mechanisms underlying ‘at risk’ pathways to psychopathology and factors that mediate this risk.
